# Supplementary material for: Environmental variation structures northern peatland soil microbiome composition and function in a reindeer herding area exclosure experiment
Source: FEMS Microbiol Ecol. 2026 Jul 8;102(8):fiag072. doi: 10.1093/femsec/fiag072 (PMC13377647; doi:10.1093/femsec/fiag072)
Supplement: fiag072_Supplemental_Files [file fiag072_supplemental_files.zip › Supplementary_Tables_Supplementary_Data.pdf]

Supplementary Table 1. Differentially expressed (false discovery rate ≤ 0.1) taxa in the metatranscriptomics taxonomic data at the order level associated with the exclusion treatment (outside vs. inside) according to the used linear mixed effects models.

	Coefficient	P-value	FDR
Clostridia UCG-014	-0.674	<0.001	0.011
KF-JG30-C25	1.464	<0.001	0.011
Thiotrichales	1.552	<0.001	0.064
Candidatus Staskawiczbacteria	-0.383	0.001	0.064
Lachnospirales	-0.557	0.001	0.067
Rhizobiales	-0.286	0.001	0.091

Supplementary Table 2. Differentially expressed (false discovery rate ≤ 0.1) taxa in the metatranscriptomics taxonomic data at the order level associated with snow increase (snow increase vs. control) according to the used linear mixed effects models.

	Coefficient	P-value	FDR
Thermoflexales	0.344	0.001	0.089

Supplementary Table 3. Differentially expressed (false discovery rate ≤ 0.1) taxa in the metatranscriptomics taxonomic data at the order level associated with snow decrease (snow decrease vs. control) according to the used linear mixed effects models.

	Coefficient	P-value	FDR
Woesearchaeales	-0.667	<0.001	0.013
Omnitrophales	-0.324	<0.001	0.064
Candidatus Kerfeldbacteria	-0.490	<0.001	0.064
Thermoflexales	0.375	0.001	0.064
Frankiales	0.676	0.001	0.064
053A03-B-DI-P58	-0.633	0.001	0.064
Spirochaetales	-0.483	0.001	0.064
Candidatus Buchananbacteria	-0.478	0.001	0.091

Supplementary Table 4. Differentially expressed (false discovery rate ≤ 0.1) taxa in the metatranscriptomics taxonomic data at the order level with significant interactions between the exclusion treatment and snow decrease according to the used linear mixed effects models.

	Coefficient exclusion*snow increase	Coefficient exclusion*snow decrease	P-value exclusion*snow increase	P-value exclusion*snow decrease	FDR exclusion*snow increase	FDR exclusion*snow decrease
1-20	0.296	-0.713	0.134	0.001	0.608	0.064
Archaeoglobales	1.261	2.563	0.072	0.001	0.525	0.064
PB19	0.305	-0.472	0.033	0.001	0.402	0.093

Supplementary Table 5. Differentially expressed (false discovery rate ≤ 0.1) taxa in the metatranscriptomics taxonomic data at the phylum level associated with the exclusion treatment (outside vs. inside) according to the used linear mixed effects models.

	**Coefficient**	**P-value**	**FDR**
Iainarchaeota	-0.276	<0.001	0.009
Firmicutes (A-Z)	-0.369	0.001	0.035
SAR324	-0.333	0.001	0.039

Supplementary Table 6. Differentially expressed (false discovery rate ≤ 0.1) taxa in the metatranscriptomics taxonomic data at the phylum level associated with snow decrease (snow decrease vs. control) according to the used linear mixed effects models.

	**Coefficient**	**P-value**	**FDR**
Margulisbacteria	-0.720	<0.001	0.024
Nanoarchaeota	-0.497	<0.001	0.026
Actinomycetota	0.532	<0.001	0.026
FCPU426	-0.372	0.003	0.081
Aquificota	0.453	0.003	0.081
Firestonebacteria	-0.758	0.004	0.098

Supplementary Table 7. Differentially expressed (false discovery rate ≤ 0.1) taxa in the metatranscriptomics taxonomic data at the phylum level with significant interactions between the exclusion treatment and snow decrease according to the used linear mixed effects models.

	**Coefficient exclusion*snow increase**	**Coefficient exclusion*snow decrease**	**P-value exclusion*snow increase**	**P-value exclusion*snow decrease**	**FDR exclusion*snow increase**	**FDR exclusion*snow decrease**
Abditibacteriota	-0.430	-0.993	0.099	<0.001	0.505	0.026

Supplementary Table 8. Differentially abundant (false discovery rate ≤ 0.1) KEGG orthology (KO) groups in the metagenomics KEGG KO data associated with the exclusion treatment (outside vs. inside) according to the used linear mixed effects models.

	**Coefficient**	**P-value**	**FDR**
K06181; rluE; 23S rRNA pseudouridine2457 synthase [EC:5.4.99.20]	0.222	<0.001	0.006
K19003; mgdA; 1,2-diacylglycerol 3-beta-glucosyltransferase [EC:2.4.1.336]	0.372	<0.001	0.044
K09014; sufB; Fe-S cluster assembly protein SufB	0.100	<0.001	0.044
K14441; rimO; ribosomal protein S12 methylthiotransferase [EC:2.8.4.4]	0.087	<0.001	0.044
K02986; RP-S4, NAM9, rpsD; small subunit ribosomal protein S4	0.109	<0.001	0.044
K02484; K02484; two-component system, OmpR family, sensor kinase [EC:2.7.13.3]	0.153	<0.001	0.050
K18479; yihS; sulfoquinovose isomerase [EC:5.3.1.31]	0.433	<0.001	0.050
K03090; sigB; RNA polymerase sigma-B factor	0.396	<0.001	0.050
K10544; xylH; D-xylose transport system permease protein	0.248	<0.001	0.050
K23536; nupC; general nucleoside transport system permease protein	0.187	<0.001	0.050
K06168; miaB; tRNA-2-methylthio-N6-dimethylallyladenosine synthase [EC:2.8.4.3]	0.086	<0.001	0.050
K09015; sufD; Fe-S cluster assembly protein SufD	0.101	<0.001	0.050
K21471; cwlO; peptidoglycan DL-endopeptidase CwlO [EC:3.4.-.-]	0.286	<0.001	0.058
K16329; psuG; pseudouridylate synthase [EC:4.2.1.70]	0.190	<0.001	0.063
K08093; hxlA; 3-hexulose-6-phosphate synthase [EC:4.1.2.43]	0.295	<0.001	0.066
K02871; RP-L13, MRPL13, rplM; large subunit ribosomal protein L13	0.085	<0.001	0.089
K00331; nuoB; NADH-quinone oxidoreductase subunit B [EC:7.1.1.2]	0.080	<0.001	0.099
K20327; xagB; glycosyltransferase XagB	0.405	<0.001	0.099
K18955; whiB1_2_3_4; WhiB family transcriptional regulator, redox-sensing transcriptional regulator	0.436	<0.001	0.099
K03867; BGLUT; UDP-glucose:tetrahydrobiopterin glucosyltransferase [EC:2.4.1.-]	0.627	<0.001	0.099
K09013; sufC; Fe-S cluster assembly ATP-binding protein	0.124	<0.001	0.099
K07695; devR; two-component system, NarL family, response regulator DevR	0.310	<0.001	0.099
K00339; nuoJ; NADH-quinone oxidoreductase subunit J [EC:7.1.1.2]	0.084	<0.001	0.099
K03076; secY; preprotein translocase subunit SecY	0.092	<0.001	0.099
K01609; trpC; indole-3-glycerol phosphate synthase [EC:4.1.1.48]	0.087	<0.001	0.099
K00423; E1.10.3.3; L-ascorbate oxidase [EC:1.10.3.3]	-0.477	<0.001	0.099

Supplementary Table 9. Differentially expressed (false discovery rate ≤ 0.1) KEGG orthology (KO) groups in the metatranscriptomics KEGG KO data associated with the exclusion treatment (outside vs. inside) according to the used linear mixed effects models.

	**Coefficient**	**P-value**	**FDR**
K00548; metH, MTR; 5-methyltetrahydrofolate--homocysteine methyltransferase [EC:2.1.1.13]	0.255	<0.001	0.032

Supplementary Table 10. Differentially abundant (false discovery rate ≤ 0.1) KEGG orthology (KO) groups in the metagenomics KEGG KO data associated with snow decrease (outside vs. inside) according to the used linear mixed effects models.

	Coefficient	P-value	FDR
K03462; NAMPT; nicotinamide phosphoribosyltransferase [EC:2.4.2.12]	-0.555	<0.001	0.044

Supplementary Table 11. Differentially expressed (false discovery rate ≤ 0.1) KEGG orthology (KO) groups in the metatranscriptomics KEGG KO data associated with snow decrease (snow decrease vs. control) according to the used linear mixed effects models.

	Coefficient	P-value	FDR
K13195; CIRBP; cold-inducible RNA-binding protein	-0.706	<0.001	0.032

Supplementary Table 12. The enriched KEGG modules in metagenomics data associated with the exclusion treatment (outside vs. inside) according to gene set enrichment analysis (GSEA) performed on the direction-adjusted significance scores (1 – p-value) from the used linear mixed effects models.

Module	P-value	BH adjusted P-value	Log2err	Enrichment score	Normalized enrichment score	Module size
M00529_Denitrification, nitrate => nitrogen	<0.001	0.012	0.498	-0.712	-2.459	10
M00567_Methanogenesis, CO2 => methane	0.001	0.025	0.455	0.483	1.856	29

Supplementary Table 13. The enriched KEGG modules in metagenomics data associated with snow increase (snow increase vs. control)) according to gene set enrichment analysis (GSEA) performed on the direction-adjusted significance scores (1 – p-value) from the used linear mixed effects models.

Module	P-value	BH adjusted P-value	Log2err	Enrichment score	Normalized enrichment score	Module size
M00174_Methane oxidation, methanotroph, methane => formaldehyde	<0.001	<0.001	0.611	-0.847	-2.235	10
M00804_Complete nitrification, comammox, ammonia => nitrite => nitrate	0.001	0.019	0.455	-0.824	-1.844	6
M00378_F420 biosynthesis, archaea	0.001	0.019	0.455	0.890	1.950	5
M00567_Methanogenesis, CO2 => methane	0.002	0.025	0.432	0.478	1.787	29
M00935_Methanofuran biosynthesis	0.004	0.037	0.407	0.786	1.816	6
M00596_Dissimilatory sulfate reduction, sulfate => H2S	0.009	0.063	0.381	-0.649	-1.768	11
M00528_Nitrification, ammonia => nitrite	0.011	0.069	0.381	-0.834	-1.631	4.000

Supplementary Table 14. The enriched KEGG modules in metagenomics data associated with snow decrease (snow decrease vs. control)) according to gene set enrichment analysis (GSEA) performed on the direction-adjusted significance scores (1 – p-value) from the used linear mixed effects models.

Module	P-value	BH adjusted P-value	Log2err	Enrichment score	Normalized enrichment score	Module size
M00378_F420 biosynthesis, archaea	0.001	0.027	0.477	0.884	2.036	5
M00567_Methanogenesis, CO2 => methane	0.001	0.027	0.455	0.476	1.934	29
M00009_Citrate cycle (TCA cycle, Krebs cycle)	0.005	0.070	0.407	-0.480	-1.731	32
M00002_Glycolysis, core module involving three-carbon compounds	0.006	0.071	0.407	-0.655	-1.775	11.000
M00308_Semi-phosphorylative Entner-Doudoroff pathway, gluconate => glycerate-3P	0.008	0.072	0.381	-0.744	-1.652	6.000

Supplementary Table 15. The enriched KEGG modules in metatranscriptomics data associated with the exclusion treatment (outside vs. inside) according to gene set enrichment analysis (GSEA) performed on the direction-adjusted significance scores (1 – p-value) from the used linear mixed effects models.

Module	P-value	BH adjusted P-value	Log2err	Enrichment score	Normalized enrichment score	Module size
M00579_Phosphate acetyltransferase-acetate kinase pathway, acetyl-CoA => acetate	0.001	0.033	0.477	-0.894	-1.975	4
M00003_Gluconeogenesis, oxaloacetate => fructose-6P	0.002	0.033	0.455	-0.502	-2.025	19
M00374_Dicarboxylate-hydroxybutyrate cycle	0.005	0.046	0.407	-0.490	-1.876	17
M00529_Denitrification, nitrate => nitrogen	0.005	0.046	0.407	-0.707	-2.050	8
M00567_Methanogenesis, CO2 => methane	0.006	0.046	0.407	0.472	1.706	27
M00307_Pyruvate oxidation, pyruvate => acetyl-CoA	0.007	0.046	0.407	-0.587	-1.828	10.000
M00173_Reductive citrate cycle (Arnon-Buchanan cycle)	0.018	0.098	0.352	-0.347	-1.614	31.000

Supplementary Table 16. The enriched KEGG modules in metatranscriptomics data associated with snow increase (snow increase vs. control)) according to gene set enrichment analysis (GSEA) performed on the direction-adjusted significance scores (1 – p-value) from the used linear mixed effects models.

Module	P-value	BH adjusted P-value	Log2err	Enrichment score	Normalized enrichment score	Module size
M00804_Complete nitrification, comammox, ammonia => nitrite => nitrate	<0.001	0.001	0.557	-0.902	-2.052	6
M00358_Coenzyme M biosynthesis	<0.001	0.007	0.498	0.974	1.757	3
M00528_Nitrification, ammonia => nitrite	0.002	0.020	0.455	-0.901	-1.789	4
M00165_Reductive pentose phosphate cycle (Calvin cycle)	0.008	0.076	0.407	-0.564	-1.805	16

Supplementary Table 17. The enriched KEGG modules in metatranscriptomics data associated with snow decrease (snow decrease vs. control)) according to gene set enrichment analysis (GSEA) performed on the direction-adjusted significance scores (1 – p-value) from the used linear mixed effects models.

Module	P-value	BH adjusted P-value	Log2err	Enrichment score	Normalized enrichment score	Module size
M00358_Coenzyme M biosynthesis	0.001	0.032	0.477	0.954	1.760	3

Supplementary Table 18. The summary statistics of metabolic marker gene ratios associated with methane oxidation to methanogenesis (*pmoa* / *mcrA*), ammonification to denitrification (*nrfA* - (*nirK* + *nirS*) and N_2O production to N_2O reduction (*nirK* + *nirS*) / *nosZ* in metagenomics and metatranscriptomics data.

	Metagenomics			Metatranscriptomics		
	mcrA / pmoA	***nrfA - (nirK + nirS)***	***(nirK + nirS) / nosZ***	***mcrA / pmoA***	***nrfA - (nirK + nirS)***	***(nirK + nirS) / nosZ***
**Min.**	0.535	-38.173	1.978	0.165	-153.681	0.946
**1st Qu.**	1.773	-28.407	2.216	0.817	-72.478	1.962
**Median**	4.267	-16.557	2.386	2.113	-50.183	2.554
**Mean**	9.028	-15.989	2.490	26.158	-55.014	2.920
**3rd Qu.**	11.274	-5.858	2.658	11.845	-32.646	3.253
**Max.**	55.173	43.175	3.511	684.795	2.282	8.024

Supplementary Table 19. The distribution of vegetation clusters across the exclusion treatment and the snow manipulation treatments.

	Trichophorum cespitosum	***Carex chordorrhiza***	***Carex rostrata***
**Inside exclosure**	14	4	0
**Outside exclosure**	6	2	10
**Snow ambient (AMB)**	8	2	2
**Snow addition (+S)**	5	3	4
**Snow removal (-S)**	7	1	4

Supplementary Table 20. The summary statistics for key variables for the 113 medium- and high-quality metagenome assembled genomes (MAGs) in the Puukkosuo metagenomics data.

	Minimum	First quartile	Median	Mean	Third quartile	Maximum
Total length	518458	1920180	2809684	2781683.0	3328616	6978053
Number of contigs	108	417	534	560.5	668	1441
N50 of the contigs	3200	3961	4667	6069.6	5851	59884
GC content	38.9	55.1	60.3	59.4	64.6	70.5
Completion (%)	50.7	57.7	67.6	69.5	78.9	98.6
Redundandy (%)	0	1.4	4.2	4.6	7.0	9.9

Supplementary Table 21. Individual metrics and taxonomic assignments for the 113 medium- and high-quality metagenome assembled genomes (MAGs) in the Puukkosuo metagenomics data. Metagenomic reads were co-assembled separately for sample plots outside and inside the exclosure with MEGAHIT, processed in anvi'o, and binned with MetaBAT2, followed by manual refinement to MIMAG standards (≥ 50 % completeness, < 10 % redundancy). MAGs were dereplicated using FastANI, and taxonomy was assigned with GTDB-Tk.

MAG name; Phylum; Order; Genus	Total length	Number of contigs	N50 of the contigs	GC content	Completion (%)	Redundancy (%)	Domain	Phylum	Class	Order	Family	Genus	Species
MAG1; Nitrospirota; Thermodesulfovibrionales; GW-Nitrospira-1	1715042	335	5445	47.2	77.5	5.6	*Bacteria*	*Nitrospirota*	*Thermodesulfovibrionia*	*Thermodesulfovibrionales*	*UBA6898*	*GW-Nitrospira-1*	
MAG2; Electryoneota; RPQS01;	1369625	350	3976	57.5	52.1	0.0	*Bacteria*	*Electryoneota*	*RPQS01*	*RPQS01*	*RPQS01*		
MAG3; Nitrospirota; Thermodesulfovibrionales; UBA6898	2930882	280	17269	49.5	91.5	7.0	*Bacteria*	*Nitrospirota*	*Thermodesulfovibrionia*	*Thermodesulfovibrionales*	*UBA6898*	*UBA6898*	
MAG4; Chloroflexota; Anaerolineales; DYJB01	2664205	595	4593	66.1	64.8	4.2	*Bacteria*	*Chloroflexota*	*Anaerolineae*	*Anaerolineales*	*E44-bin32*	*DYJB01*	
MAG5; Verrucomicrobiota; Chthoniobacterales; Terrimicrobium	2522620	604	4255	58.4	71.8	4.2	*Bacteria*	*Verrucomicrobiota*	*Verrucomicrobiae*	*Chthoniobacterales*	*Terrimicrobiaceae*	*Terrimicrobium*	
MAG6; Pseudomonadota; Burkholderiales;	2475112	534	4865	59.7	76.1	0.0	*Bacteria*	*Pseudomonadota*	*Gammaproteobacteria*	*Burkholderiales*	*Usitatibacteraceae*		
MAG7; Pseudomonadota; Pseudomonadales; SZUA-521	2010477	481	4063	61.6	56.3	1.4	*Bacteria*	*Pseudomonadota*	*Gammaproteobacteria*	*Pseudomonadales*	*Azotimanducaceae_A*	*SZUA-521*	
MAG8; Actinomycetota; IMCC26256; JALHSW01	1266938	333	3724	69.3	57.7	8.5	*Bacteria*	*Actinomycetota*	*Acidimicrobiia*	*IMCC26256*	*PALSA-555*	*JALHSW01*	
MAG9; Acidobacteriota; Fen-336;	1879369	514	3584	67.7	66.2	2.8	*Bacteria*	*Acidobacteriota*	*Vicinamibacteria*	*Fen-336*	*Fen-336*		
MAG10; Methylomirabilota; Rokubacteriales; AR37	3839230	790	5085	67.5	91.5	5.6	*Bacteria*	*Methylomirabilota*	*Methylomirabilia*	*Rokubacteriales*	*CSP1-6*	*AR37*	
MAG11; Actinomycetota; UBA4738; AC-51	1457159	375	3858	68.2	64.8	2.8	*Bacteria*	*Actinomycetota*	*UBA4738*	*UBA4738*	*HRBIN12*	*AC-51*	
MAG12; Pseudomonadota; Burkholderiales;	1867187	469	3931	63.1	62.0	8.5	*Bacteria*	*Pseudomonadota*	*Gammaproteobacteria*	*Burkholderiales*	*Burkholderiaceae*		
MAG13; Bacteroidota; Bacteroidales; LD21	1896446	473	4094	41.4	50.7	0.0	*Bacteria*	*Bacteroidota*	*Bacteroidia*	*Bacteroidales*	*VadinHA17*	*LD21*	
MAG14; Pseudomonadota; Burkholderiales; Sideroxyarcus	1738360	433	4012	55.6	64.8	4.2	*Bacteria*	*Pseudomonadota*	*Gammaproteobacteria*	*Burkholderiales*	*Gallionellaceae*	*Sideroxyarcus*	
MAG15; Eisenbacteria; RBG-16-71-46; WS-11	711033	206	3320	68.0	53.5	0.0	*Bacteria*	*Eisenbacteria*	*RBG-16-71-46*	*RBG-16-71-46*	*RBG-16-71-46*	*WS-11*	
MAG16; Pseudomonadota; Dongiales;	2809684	646	4356	63.5	56.3	9.9	*Bacteria*	*Pseudomonadota*	*Alphaproteobacteria*	*Dongiales*	*Dongiaceae*		
MAG17; Pseudomonadota; Burkholderiales;	3370801	607	5851	60.3	94.4	4.2	*Bacteria*	*Pseudomonadota*	*Gammaproteobacteria*	*Burkholderiales*	*Usitatibacteraceae*		
MAG18; Methylomirabilota; Rokubacteriales;	2730710	718	3676	67.7	54.9	1.4	*Bacteria*	*Methylomirabilota*	*Methylomirabilia*	*Rokubacteriales*	*CSP1-6*		
MAG19; Nitrospirota; Nitrospirales; Palsa-1315	3474959	323	17969	56.7	95.8	1.4	*Bacteria*	*Nitrospirota*	*Nitrospiria*	*Nitrospirales*	*Nitrospiraceae*	*Palsa-1315*	
MAG20; Chloroflexota; Anaerolineales; VGNF01	3127218	633	5229	55.1	77.5	2.8	*Bacteria*	*Chloroflexota*	*Anaerolineae*	*Anaerolineales*	*Villgracilaceae*	*VGNF01*	
MAG21; Nitrospirota; UBA9217; JAJYKN01	2227524	471	4796	53.4	67.6	2.8	*Bacteria*	*Nitrospirota*	*UBA9217*	*UBA9217*	*UBA9217*	*JAJYKN01*	
MAG22; Nitrospirota; Nitrospirales; Palsa-1315	3094438	447	8403	57.1	80.3	0.0	*Bacteria*	*Nitrospirota*	*Nitrospiria*	*Nitrospirales*	*Nitrospiraceae*	*Palsa-1315*	
MAG23; Pseudomonadota; Burkholderiales; SG8-41	3032004	642	4771	64.4	93.0	4.2	*Bacteria*	*Pseudomonadota*	*Gammaproteobacteria*	*Burkholderiales*	*SG8-41*	*SG8-41*	
MAG24; Pseudomonadota; Rhizobiales; Methyloceanibacter	1481764	377	3905	63.5	52.1	2.8	*Bacteria*	*Pseudomonadota*	*Alphaproteobacteria*	*Rhizobiales*	*Methyloligellaceae*	*Methyloceanibacter*	
MAG25; Pseudomonadota; Rhizobiales; Methylocystis	3840714	593	7862	61.5	93.0	0.0	*Bacteria*	*Pseudomonadota*	*Alphaproteobacteria*	*Rhizobiales*	*Beijerinckiaceae*	*Methylocystis*	*Methylocystis sp021731785*
MAG26; Nitrospirota; UBA9217; JAJYKN01	2144969	534	3981	57.4	62.0	9.9	*Bacteria*	*Nitrospirota*	*UBA9217*	*UBA9217*	*UBA9217*	*JAJYKN01*	
MAG27; Nitrospirota; Nitrospirales; Palsa-1315	2843453	375	10165	56.3	87.3	4.2	*Bacteria*	*Nitrospirota*	*Nitrospiria*	*Nitrospirales*	*Nitrospiraceae*	*Palsa-1315*	
MAG28; Desulfobacterota; Desulfomonilales;	3873733	951	4080	53.1	57.7	8.5	*Bacteria*	*Desulfobacterota*	*Desulfomonilia*	*Desulfomonilales*	*Desulfomonilaceae*		
MAG29; Chloroflexota; Anaerolineales; Defluviilinea	1482676	387	3799	53.2	60.6	0.0	*Bacteria*	*Chloroflexota*	*Anaerolineae*	*Anaerolineales*	*Villgracilaceae*	*Defluviilinea*	
MAG30; Pseudomonadota; Burkholderiales; JALORV01	3282903	672	5095	63.4	70.4	4.2	*Bacteria*	*Pseudomonadota*	*Gammaproteobacteria*	*Burkholderiales*	*Burkholderiaceae*	*JALORV01*	
MAG31; Methylomirabilota; Rokubacteriales; AR37	4429943	1044	4372	67.3	54.9	7.0	*Bacteria*	*Methylomirabilota*	*Methylomirabilia*	*Rokubacteriales*	*CSP1-6*	*AR37*	
MAG32; Desulfobacterota; Geobacterales; CAIPTY01	3311607	696	4956	56.5	70.4	5.6	*Bacteria*	*Desulfobacterota*	*Desulfuromonadia*	*Geobacterales*	*Pseudopelobacteraceae*	*CAIPTY01*	
MAG33; Chloroflexota; Anaerolineales; Defluviilinea	1920180	444	4326	56.7	60.6	4.2	*Bacteria*	*Chloroflexota*	*Anaerolineae*	*Anaerolineales*	*Villgracilaceae*	*Defluviilinea*	
MAG34; Bacteroidota; Bacteroidales; LD21	2540130	673	3615	39.7	53.5	8.5	*Bacteria*	*Bacteroidota*	*Bacteroidia*	*Bacteroidales*	*VadinHA17*	*LD21*	
MAG35; Myxococcota_A; UBA9160; PR03	1559847	410	3704	67.8	59.2	0.0	*Bacteria*	*Myxococcota_A*	*UBA9160*	*UBA9160*	*PR03*	*PR03*	
MAG36; Actinomycetota; Acidimicrobiales; JAENVT01	3931835	780	5382	64.5	77.5	4.2	*Bacteria*	*Actinomycetota*	*Acidimicrobiia*	*Acidimicrobiales*	*Ilumatobacteraceae*	*JAENVT01*	
MAG37; Myxococcota; Myxococcales; Anaeromyxobacter	2059703	454	4667	70.5	64.8	1.4	*Bacteria*	*Myxococcota*	*Myxococcia*	*Myxococcales*	*Anaeromyxobacteraceae*	*Anaeromyxobacter*	
MAG38; Actinomycetota; IMCC26256;	1518780	442	3333	68.5	50.7	9.9	*Bacteria*	*Actinomycetota*	*Acidimicrobiia*	*IMCC26256*	*PALSA-555*		
MAG39; Pseudomonadota; Burkholderiales; SG8-41	3016676	622	5108	64.4	90.1	5.6	*Bacteria*	*Pseudomonadota*	*Gammaproteobacteria*	*Burkholderiales*	*SG8-41*	*SG8-41*	
MAG40; Methylomirabilota; Methylomirabilales; 2-02-FULL-66-22	5618893	1030	6020	64.1	87.3	2.8	*Bacteria*	*Methylomirabilota*	*Methylomirabilia*	*Methylomirabilales*	*2-02-FULL-66-22*	*2-02-FULL-66-22*	
MAG41; Desulfobacterota_B; UBA9968; DP-20	2842652	668	4339	57.3	74.6	5.6	*Bacteria*	*Desulfobacterota_B*	*Binatia*	*UBA9968*	*UBA9968*	*DP-20*	
MAG42; Pseudomonadota; Rhizobiales; Rhodoplanes	2931872	610	5049	64.6	74.6	5.6	*Bacteria*	*Pseudomonadota*	*Alphaproteobacteria*	*Rhizobiales*	*Xanthobacteraceae*	*Rhodoplanes*	
MAG43; Pseudomonadota; Steroidobacterales; CADEED01	2014806	351	6385	64.3	78.9	1.4	*Bacteria*	*Pseudomonadota*	*Gammaproteobacteria*	*Steroidobacterales*	*Steroidobacteraceae*	*CADEED01*	
MAG44; Desulfobacterota_B; DP-6;	1717869	497	3297	68.2	50.7	1.4	*Bacteria*	*Desulfobacterota_B*	*Binatia*	*DP-6*	*DP-6*		
MAG45; Desulfobacterota_B; UBA9968; DP-1	4216331	687	6878	54.2	90.1	5.6	*Bacteria*	*Desulfobacterota_B*	*Binatia*	*UBA9968*	*UBA9968*	*DP-1*	
MAG46; Actinomycetota; UBA5794; JALYNX01	1499894	381	3910	62.7	67.6	4.2	*Bacteria*	*Actinomycetota*	*Acidimicrobiia*	*UBA5794*	*ZC4RG35*	*JALYNX01*	
MAG47; Chloroflexota; Anaerolineales; VGNF01	2947147	603	5156	56.5	78.9	1.4	*Bacteria*	*Chloroflexota*	*Anaerolineae*	*Anaerolineales*	*Villgracilaceae*	*VGNF01*	
MAG48; Verrucomicrobiota; Chthoniobacterales; Terrimicrobium	2468182	627	3871	58.4	62.0	5.6	*Bacteria*	*Verrucomicrobiota*	*Verrucomicrobiae*	*Chthoniobacterales*	*Terrimicrobiaceae*	*Terrimicrobium*	
MAG49; Actinomycetota; Acidimicrobiales; JAENVT01	2973888	656	4673	65.6	84.5	5.6	*Bacteria*	*Actinomycetota*	*Acidimicrobiia*	*Acidimicrobiales*	*Ilumatobacteraceae*	*JAENVT01*	
MAG50; Methylomirabilota; Rokubacteriales; AR37	2516603	467	6060	67.6	67.6	8.5	*Bacteria*	*Methylomirabilota*	*Methylomirabilia*	*Rokubacteriales*	*CSP1-6*	*AR37*	
MAG51; Pseudomonadota; Burkholderiales; 2-12-FULL-64-23	4826209	786	7191	63.1	97.2	0.0	*Bacteria*	*Pseudomonadota*	*Gammaproteobacteria*	*Burkholderiales*	*SG8-39*	*2-12-FULL-64-23*	
MAG52; Chloroflexota; UBA4142;	575945	173	3200	53.0	50.7	2.8	*Bacteria*	*Chloroflexota*	*Anaerolineae*	*UBA4142*	*UBA4142*		
MAG53; Chloroflexota; Anaerolineales; Defluviilinea	3264859	450	8684	53.9	76.1	4.2	*Bacteria*	*Chloroflexota*	*Anaerolineae*	*Anaerolineales*	*Villgracilaceae*	*Defluviilinea*	
MAG54; Methylomirabilota; Rokubacteriales; CAMLFJ01	1526534	427	3494	68.0	50.7	4.2	*Bacteria*	*Methylomirabilota*	*Methylomirabilia*	*Rokubacteriales*	*CSP1-6*	*CAMLFJ01*	
MAG55; Pseudomonadota; Burkholderiales; 2-12-FULL-64-23	3924542	645	7136	63.0	87.3	7.0	*Bacteria*	*Pseudomonadota*	*Gammaproteobacteria*	*Burkholderiales*	*SG8-39*	*2-12-FULL-64-23*	
MAG56; Chloroflexota; Anaerolineales; Villigracilis	3064206	604	5588	48.9	69.0	1.4	*Bacteria*	*Chloroflexota*	*Anaerolineae*	*Anaerolineales*	*Villgracilaceae*	*Villigracilis*	
MAG57; Nitrospirota; UBA9217; JAAXXJ01	2893875	440	7812	58.3	76.1	4.2	*Bacteria*	*Nitrospirota*	*UBA9217*	*UBA9217*	*UBA9217*	*JAAXXJ01*	
MAG58; Nitrospirota; Nitrospirales; Palsa-1315	3328616	223	25662	56.1	98.6	0.0	*Bacteria*	*Nitrospirota*	*Nitrospiria*	*Nitrospirales*	*Nitrospiraceae*	*Palsa-1315*	
MAG59; Bacteroidota; Bacteroidales; LD21	3233407	485	7991	38.9	77.5	1.4	*Bacteria*	*Bacteroidota*	*Bacteroidia*	*Bacteroidales*	*VadinHA17*	*LD21*	
MAG60; Desulfobacterota_E; Deferrimicrobiales; Deferrimicrobium	1632087	351	4998	66.7	69.0	9.9	*Bacteria*	*Desulfobacterota_E*	*Deferrimicrobia*	*Deferrimicrobiales*	*Deferrimicrobiaceae*	*Deferrimicrobium*	
MAG61; Chloroflexota; Anaerolineales; Defluviilinea	1179682	339	3351	53.3	50.7	7.0	*Bacteria*	*Chloroflexota*	*Anaerolineae*	*Anaerolineales*	*Villgracilaceae*	*Defluviilinea*	
MAG62; Pseudomonadota; Rhizobiales; Methyloceanibacter	2982805	834	3402	62.6	53.5	8.5	*Bacteria*	*Pseudomonadota*	*Alphaproteobacteria*	*Rhizobiales*	*Methyloligellaceae*	*Methyloceanibacter*	
MAG63; Actinomycetota; UBA4738; AC-51	1452837	338	4288	68.6	62.0	1.4	*Bacteria*	*Actinomycetota*	*UBA4738*	*UBA4738*	*HRBIN12*	*AC-51*	
MAG64; Chloroflexota; Anaerolineales; Defluviilinea	5092441	839	6830	53.4	76.1	8.5	*Bacteria*	*Chloroflexota*	*Anaerolineae*	*Anaerolineales*	*Villgracilaceae*	*Defluviilinea*	
MAG65; Chloroflexota; Anaerolineales; VGNF01	3479450	465	9228	51.0	81.7	2.8	*Bacteria*	*Chloroflexota*	*Anaerolineae*	*Anaerolineales*	*Villgracilaceae*	*VGNF01*	
MAG66; Acidobacteriota; Pyrinomonadales; UBA11740	3364690	789	4331	56.6	50.7	8.5	*Bacteria*	*Acidobacteriota*	*Blastocatellia*	*Pyrinomonadales*	*Pyrinomonadaceae*	*UBA11740*	
MAG67; Nitrospirota; UBA9217; JAJYKN01	1984827	463	4271	53.4	60.6	4.2	*Bacteria*	*Nitrospirota*	*UBA9217*	*UBA9217*	*UBA9217*	*JAJYKN01*	
MAG68; Pseudomonadota; Burkholderiales; JAEUMW01	1612268	436	3605	64.6	62.0	0.0	*Bacteria*	*Pseudomonadota*	*Gammaproteobacteria*	*Burkholderiales*	*JAEUMW01*	*JAEUMW01*	
MAG69; Chloroflexota; Anaerolineales; UBA700	2644304	680	3863	54.1	67.6	1.4	*Bacteria*	*Chloroflexota*	*Anaerolineae*	*Anaerolineales*	*Anaerolineaceae*	*UBA700*	
MAG70; Chloroflexota; Anaerolineales; VGNF01	3506739	582	7070	50.5	57.7	7.0	*Bacteria*	*Chloroflexota*	*Anaerolineae*	*Anaerolineales*	*Villgracilaceae*	*VGNF01*	
MAG71; Pseudomonadota; Rhizobiales; Hyphomicrobium_A	3106484	653	4866	62.9	62.0	8.5	*Bacteria*	*Pseudomonadota*	*Alphaproteobacteria*	*Rhizobiales*	*Hyphomicrobiaceae*	*Hyphomicrobium_A*	
MAG72; Bacteroidota; Bacteroidales; LD21	2899426	556	5490	41.0	69.0	9.9	*Bacteria*	*Bacteroidota*	*Bacteroidia*	*Bacteroidales*	*VadinHA17*	*LD21*	
MAG73; Bacteroidota; Bacteroidales; LD21	3252435	399	9887	39.2	70.4	5.6	*Bacteria*	*Bacteroidota*	*Bacteroidia*	*Bacteroidales*	*VadinHA17*	*LD21*	
MAG74; Nitrospirota; UBA9217; JALNZF01	1327174	325	4286	54.6	54.9	2.8	*Bacteria*	*Nitrospirota*	*UBA9217*	*UBA9217*	*UBA9217*	*JALNZF01*	
MAG75; Pseudomonadota; Burkholderiales; CAISUK01	2263784	519	4401	62.1	70.4	4.2	*Bacteria*	*Pseudomonadota*	*Gammaproteobacteria*	*Burkholderiales*	*Rhodocyclaceae*	*CAISUK01*	
MAG76; Nitrospirota; Thermodesulfovibrionales; JAJYIF01	3253218	108	59884	49.9	94.4	1.4	*Bacteria*	*Nitrospirota*	*Thermodesulfovibrionia*	*Thermodesulfovibrionales*	*UBA9159*	*JAJYIF01*	
MAG77; Chloroflexota; Anaerolineales; Villigracilis	2469219	575	4252	49.1	53.5	5.6	*Bacteria*	*Chloroflexota*	*Anaerolineae*	*Anaerolineales*	*Villgracilaceae*	*Villigracilis*	
MAG78; Acidobacteriota; Fen-336;	4600401	809	6582	68.2	87.3	8.5	*Bacteria*	*Acidobacteriota*	*Vicinamibacteria*	*Fen-336*	*Fen-336*		
MAG79; Actinomycetota; Gaiellales; GMQP-bins7	1338404	400	3244	67.8	52.1	9.9	*Bacteria*	*Actinomycetota*	*Thermoleophilia*	*Gaiellales*	*Gaiellaceae*	*GMQP-bins7*	
MAG80; Desulfobacterota_B; UBA9968; JACPFE01	3358669	717	5018	56.7	60.6	5.6	*Bacteria*	*Desulfobacterota_B*	*Binatia*	*UBA9968*	*UBA9968*	*JACPFE01*	
MAG81; Pseudomonadota; Burkholderiales;	4098068	634	7656	63.5	87.3	1.4	*Bacteria*	*Pseudomonadota*	*Gammaproteobacteria*	*Burkholderiales*	*Burkholderiaceae*		
MAG82; Desulfobacterota_B; HRBIN30; JAKLJW01	4445937	1101	4021	66.6	76.1	8.5	*Bacteria*	*Desulfobacterota_B*	*Binatia*	*HRBIN30*	*JAGDMS01*	*JAKLJW01*	
MAG83; Actinomycetota; Gaiellales; GMQP-bins7	1970417	417	4962	68.0	74.6	4.2	*Bacteria*	*Actinomycetota*	*Thermoleophilia*	*Gaiellales*	*Gaiellaceae*	*GMQP-bins7*	
MAG84; Pseudomonadota; Rariloculales; FEN-1219	2320846	552	4315	65.2	64.8	1.4	*Bacteria*	*Pseudomonadota*	*Gammaproteobacteria*	*Rariloculales*	*Rariloculaceae*	*FEN-1219*	
MAG85; Pseudomonadota; Rhizobiales; Rhodoplanes	3662794	763	5074	63.6	59.2	2.8	*Bacteria*	*Pseudomonadota*	*Alphaproteobacteria*	*Rhizobiales*	*Xanthobacteraceae*	*Rhodoplanes*	
MAG86; Pseudomonadota; Dongiales;	4235489	548	9404	64.0	87.3	5.6	*Bacteria*	*Pseudomonadota*	*Alphaproteobacteria*	*Dongiales*	*Dongiaceae*		
MAG87; Acidobacteriota; Pyrinomonadales; JAOAPY01	6978053	672	13743	56.4	98.6	8.5	*Bacteria*	*Acidobacteriota*	*Blastocatellia*	*Pyrinomonadales*	*Pyrinomonadaceae*	*JAOAPY01*	
MAG88; Methylomirabilota; Rokubacteriales;	4526313	1158	3868	67.1	54.9	9.9	*Bacteria*	*Methylomirabilota*	*Methylomirabilia*	*Rokubacteriales*	*CSP1-6*		
MAG89; Acidobacteriota; Fen-336;	4620181	1054	4443	68.8	83.1	5.6	*Bacteria*	*Acidobacteriota*	*Vicinamibacteria*	*Fen-336*	*Fen-336*		
MAG90; Acidobacteriota; Pyrinomonadales; PSRF01	6547371	733	11808	55.1	93.0	4.2	*Bacteria*	*Acidobacteriota*	*Blastocatellia*	*Pyrinomonadales*	*Pyrinomonadaceae*	*PSRF01*	
MAG91; Bacteroidota; Bacteroidales; LD21	2914866	642	4706	41.9	80.3	5.6	*Bacteria*	*Bacteroidota*	*Bacteroidia*	*Bacteroidales*	*VadinHA17*	*LD21*	
MAG92; Tectomicrobia; Entotheonellales;	5099274	1441	3432	58.9	50.7	7.0	*Bacteria*	*Tectomicrobia*	*Entotheonellia*	*Entotheonellales*			
MAG93; Nitrospirota; Thermodesulfovibrionales; GW-Nitrospira-1	1796401	362	5352	46.8	69.0	7.0	*Bacteria*	*Nitrospirota*	*Thermodesulfovibrionia*	*Thermodesulfovibrionales*	*UBA6898*	*GW-Nitrospira-1*	
MAG94; Pseudomonadota; Competibacterales; CAIMUS01	1989426	513	3915	57.6	57.7	4.2	*Bacteria*	*Pseudomonadota*	*Gammaproteobacteria*	*Competibacterales*	*Competibacteraceae*	*CAIMUS01*	
MAG95; Tectomicrobia; J063;	3300993	866	3752	58.7	59.2	2.8	*Bacteria*	*Tectomicrobia*	*Entotheonellia*	*J063*			
MAG96; Chloroflexota; Anaerolineales; Defluviilinea	1699598	411	4220	57.2	64.8	0.0	*Bacteria*	*Chloroflexota*	*Anaerolineae*	*Anaerolineales*	*Villgracilaceae*	*Defluviilinea*	
MAG97; Pseudomonadota; Rhizobiales; Hyphomicrobium_A	3306998	689	5042	63.2	73.2	7.0	*Bacteria*	*Pseudomonadota*	*Alphaproteobacteria*	*Rhizobiales*	*Hyphomicrobiaceae*	*Hyphomicrobium_A*	
MAG98; Chloroflexota; Anaerolineales; VGNF01	2125303	513	4179	56.5	53.5	1.4	*Bacteria*	*Chloroflexota*	*Anaerolineae*	*Anaerolineales*	*Villgracilaceae*	*VGNF01*	
MAG99; Pseudomonadota; Rhizobiales; Rhodoplanes	3031342	685	4567	64.2	70.4	2.8	*Bacteria*	*Pseudomonadota*	*Alphaproteobacteria*	*Rhizobiales*	*Xanthobacteraceae*	*Rhodoplanes*	
MAG100; Pseudomonadota; Burkholderiales; Fen-999	518458	154	3299	62.9	50.7	8.5	*Bacteria*	*Pseudomonadota*	*Gammaproteobacteria*	*Burkholderiales*	*Rhodocyclaceae*	*Fen-999*	
MAG101; Pseudomonadota; Burkholderiales; JALORV01	3517878	553	7554	63.4	87.3	9.9	*Bacteria*	*Pseudomonadota*	*Gammaproteobacteria*	*Burkholderiales*	*Burkholderiaceae*	*JALORV01*	
MAG102; Methylomirabilota; Rokubacteriales; AR37	1679482	314	5534	67.8	50.7	7.0	*Bacteria*	*Methylomirabilota*	*Methylomirabilia*	*Rokubacteriales*	*CSP1-6*	*AR37*	
MAG103; Bacteroidota; UBA10030; UBA6906	1548479	387	3961	58.3	66.2	5.6	*Bacteria*	*Bacteroidota*	*UBA10030*	*UBA10030*	*UBA6906*	*UBA6906*	
MAG104; Chloroflexota; Anaerolineales; DYJB01	2215591	528	4254	66.0	70.4	8.5	*Bacteria*	*Chloroflexota*	*Anaerolineae*	*Anaerolineales*	*E44-bin32*	*DYJB01*	
MAG105; Pseudomonadota; Rhizobiales; Methyloceanibacter	2241862	445	5505	60.9	80.3	7.0	*Bacteria*	*Pseudomonadota*	*Alphaproteobacteria*	*Rhizobiales*	*Methyloligellaceae*	*Methyloceanibacter*	
MAG106; Chloroflexota; Anaerolineales; VGNF01	3249445	401	10147	55.5	93.0	5.6	*Bacteria*	*Chloroflexota*	*Anaerolineae*	*Anaerolineales*	*Villgracilaceae*	*VGNF01*	
MAG107; Bacteroidota; UBA10030; UBA9647	3499185	749	4906	53.1	69.0	2.8	*Bacteria*	*Bacteroidota*	*UBA10030*	*UBA10030*	*UBA10030*	*UBA9647*	
MAG108; Myxococcota; Haliangiales;	2225521	575	3850	68.8	59.2	5.6	*Bacteria*	*Myxococcota*	*Polyangia*	*Haliangiales*	*SHYZ01*		
MAG109; Desulfobacterota_B; HRBIN30;	2091254	581	3508	66.8	52.1	1.4	*Bacteria*	*Desulfobacterota_B*	*Binatia*	*HRBIN30*	*CADEER01*		
MAG110; Pseudomonadota; Rhizobiales; Aestuariivirga	2792052	516	6180	56.0	71.8	1.4	*Bacteria*	*Pseudomonadota*	*Alphaproteobacteria*	*Rhizobiales*	*Aestuariivirgaceae*	*Aestuariivirga*	
MAG111; Nitrospirota; UBA9217; JAAXXJ01	2056699	501	4167	58.3	52.1	4.2	*Bacteria*	*Nitrospirota*	*UBA9217*	*UBA9217*	*UBA9217*	*JAAXXJ01*	
MAG112; Chloroflexota; Anaerolineales; Defluviilinea	3041390	608	5370	52.9	77.5	5.6	*Bacteria*	*Chloroflexota*	*Anaerolineae*	*Anaerolineales*	*Villgracilaceae*	*Defluviilinea*	
MAG113; Methylomirabilota; Methylomirabilales; 2-02-FULL-66-22	2345599	573	4158	63.6	67.6	0.0	*Bacteria*	*Methylomirabilota*	*Methylomirabilia*	*Methylomirabilales*	*2-02-FULL-66-22*	*2-02-FULL-66-22*	

Supplementary Table 22. Additional manually identified and filtered eukaryotic proteins from the Compiled Greening Lab metabolic marker gene database.

**Manually identified and supplemented eukaryotic proteins**
AclB-XP_003613199.1 - Medicago truncatula
SdhA_FrdA-Arabidopsis thaliana (Group 5c)
SdhA_FrdA-Ascaris suum (Group 5c)
SdhA_FrdA-Aspergillus niger (Group 5c)
SdhA_FrdA-Blattella germanic (Group 1c)
SdhA_FrdA-Caenorhabditis elegans (Group 5c)
SdhA_FrdA-Candida albicans (Group 5c)
SdhA_FrdA-Drosophila melanogaster (Group 5c)
SdhA_FrdA-Homo sapiens (Group 5c)
SdhA_FrdA-Neurospora crassa (Group 5c)
SdhA_FrdA-Penicillium chrysogenum (Group 5c)
SdhA_FrdA-Porphyra umbilicalis (Group 1a)
SdhA_FrdA-Saccharomyces cerevisiae (Group 5c)
SdhA_FrdA-Trichophyton rubrum (Group 5c)
SdhA_FrdA-Ustilago maydis (Group 5c)
NirK-XP_003067932.1 - Coccidioides posadasii
FeFe-XP_001318941.1 - Trichomonas vaginalis - [FeFe] Group A1
Sqr-XP_029646137.1 - Octopus vulgaris - Sqr II

Supplementary Table 23. Read mapping statistics for the metagenomics and metatrancriptomics data for the different metabolic functional and taxonomics alignments used in the study. The total number of reads for all samples are given in first column for metagenomics and metatranscriptomics data, respectively. The second column gives the proportion of trimmed reads passing the quality control. For metagenomics, all remaining columns give the proportion of quality controlled mate-pair combined read alignments to the trimmed reads. For metatranscriptomics, the trimmed reads are further filtered for ribosomal RNA (rRNA) given as proportions to the trimmed reads. For metatranscriptomics, all the KEGG and metabolic marker gene hits are given as proportions to the rRNA-filtered reads while the SILVA SSU rRNA counts are given as proportions to the trimmed reads.

Metagenomics

Sample	Total read pairs	Total read pairs trimmed	Total KEGG hits	Total KEGG gene with KO	Total metabolic marker gene hits	Total SILVA SSU rRNA counts
P1	25167271	98.08 %	49.24 %	30.85 %	0.71 %	0.07 %
P2	13005673	92.27 %	42.62 %	27.09 %	0.62 %	0.05 %
P3	9579647	92.37 %	41.44 %	26.17 %	0.54 %	0.06 %
P4	15748479	91.83 %	42.32 %	26.56 %	0.56 %	0.05 %
P5	20364130	93.69 %	43.20 %	26.91 %	0.58 %	0.06 %
P6	17432323	97.11 %	47.03 %	29.52 %	0.68 %	0.07 %
P7	11665137	91.93 %	43.61 %	27.65 %	0.63 %	0.05 %
P8	17001416	92.99 %	41.55 %	26.39 %	0.62 %	0.05 %
P9	13533695	91.73 %	42.93 %	27.21 %	0.63 %	0.05 %
P10	12834923	92.68 %	40.87 %	25.75 %	0.50 %	0.06 %
P11	18398807	94.98 %	44.80 %	28.26 %	0.64 %	0.06 %
P12	15463174	92.01 %	42.03 %	26.74 %	0.61 %	0.06 %
P13	15687452	95.83 %	45.97 %	28.87 %	0.62 %	0.06 %
P14	16371329	92.54 %	42.54 %	26.70 %	0.54 %	0.05 %
P15	12709520	92.27 %	43.28 %	26.80 %	0.56 %	0.04 %
P16	12035466	91.24 %	41.07 %	25.87 %	0.56 %	0.05 %
P17	22564118	97.41 %	48.38 %	30.13 %	0.69 %	0.06 %
P18	15740660	94.19 %	44.14 %	27.60 %	0.59 %	0.05 %
P19	9518922	90.65 %	42.48 %	26.60 %	0.56 %	0.05 %
P20	14856433	94.77 %	43.82 %	27.61 %	0.66 %	0.07 %
P21	20442272	97.03 %	47.33 %	29.29 %	0.65 %	0.06 %
P22	16316648	99.30 %	49.71 %	31.45 %	0.76 %	0.08 %
P23	8774224	91.04 %	43.70 %	27.01 %	0.52 %	0.04 %
P24	17761720	92.24 %	39.87 %	25.64 %	0.60 %	0.06 %
P25	25162690	98.60 %	50.13 %	30.52 %	0.67 %	0.05 %
P26	28886145	98.96 %	52.03 %	31.92 %	0.73 %	0.06 %
P27	14478034	94.46 %	46.86 %	28.84 %	0.63 %	0.05 %
P28	12544372	90.38 %	40.41 %	25.42 %	0.55 %	0.05 %
P29	14180656	94.14 %	44.02 %	27.44 %	0.62 %	0.05 %
P30	11665715	91.10 %	42.73 %	27.04 %	0.60 %	0.05 %
P31	18834258	97.90 %	49.92 %	29.97 %	0.65 %	0.06 %
P32	10610062	94.84 %	47.45 %	29.50 %	0.67 %	0.05 %
P33	19701008	94.18 %	42.33 %	26.29 %	0.58 %	0.05 %
P34	8264857	91.67 %	42.31 %	26.17 %	0.54 %	0.04 %
P35	15030324	90.44 %	41.07 %	25.54 %	0.53 %	0.04 %
P36	15462190	91.81 %	40.69 %	25.31 %	0.50 %	0.05 %

Metatranscriptomics

Sample	Total read pairs	Total read pairs trimmed	Total read pairs rRNA filtered	Total KEGG hits	Total KEGG gene with KO	Total metaboli c marker gene hits	Total SILVA SSU rRNA counts
P1	33751654	99.85 %	4.24 %	18.36 %	12.24 %	0.46 %	35.84 %
P2	34910802	99.84 %	3.87 %	17.28 %	11.23 %	0.40 %	34.65 %
P3	24329493	99.87 %	4.00 %	21.66 %	14.21 %	0.41 %	34.87 %
P4	43438804	99.88 %	4.17 %	21.08 %	13.49 %	0.46 %	35.16 %
P5	82436550	99.77 %	4.75 %	21.88 %	14.13 %	0.41 %	35.32 %
P6	50743771	99.87 %	3.98 %	18.84 %	12.37 %	0.48 %	35.89 %
P7	47603328	99.84 %	5.36 %	18.26 %	11.57 %	0.36 %	34.07 %
P8	33887176	99.89 %	5.13 %	24.64 %	16.41 %	0.84 %	35.08 %
P9	33545784	99.89 %	4.57 %	21.47 %	13.93 %	0.41 %	34.23 %
P10	30477031	99.84 %	4.31 %	20.00 %	12.60 %	0.34 %	35.12 %
P11	45862483	99.88 %	5.06 %	17.92 %	11.39 %	0.37 %	34.70 %
P12	33640735	99.88 %	5.04 %	22.38 %	14.47 %	0.42 %	34.63 %
P13	51056735	99.74 %	4.18 %	18.95 %	12.46 %	0.43 %	36.17 %
P14	29241743	99.86 %	4.79 %	19.38 %	12.08 %	0.31 %	35.70 %
P15	33944127	99.86 %	5.10 %	24.48 %	15.74 %	0.35 %	34.74 %
P16	34143578	99.87 %	3.88 %	19.98 %	12.89 %	0.33 %	35.49 %
P17	51385751	99.87 %	3.87 %	18.61 %	12.18 %	0.45 %	36.29 %
P18	39422598	99.88 %	4.63 %	20.96 %	13.57 %	0.36 %	35.23 %
P19	34140671	99.84 %	4.04 %	19.73 %	12.59 %	0.33 %	34.65 %
P20	39974397	99.88 %	4.23 %	16.77 %	10.91 %	0.48 %	35.44 %
P21	35926836	99.32 %	4.01 %	18.03 %	11.89 %	0.39 %	36.06 %
P22	26800494	99.88 %	4.56 %	18.01 %	11.95 %	0.56 %	34.67 %
P23	72336681	99.78 %	5.02 %	19.79 %	12.27 %	0.25 %	33.59 %
P24	35206478	99.87 %	4.67 %	17.81 %	11.93 %	0.57 %	33.86 %
P25	41719853	99.81 %	4.38 %	17.01 %	10.86 %	0.37 %	34.36 %
P26	31456366	99.88 %	4.00 %	18.51 %	11.72 %	0.47 %	35.71 %
P27	32189269	99.84 %	4.22 %	20.07 %	12.96 %	0.38 %	35.37 %
P28	29075206	99.81 %	5.22 %	22.39 %	14.08 %	0.34 %	35.07 %
P29	34332202	99.89 %	4.81 %	21.56 %	14.03 %	0.48 %	34.08 %
P30	26487311	99.87 %	4.42 %	23.56 %	15.22 %	0.38 %	35.55 %
P31	27396219	99.87 %	4.35 %	20.99 %	12.87 %	0.38 %	36.01 %
P32	34915485	99.88 %	4.31 %	19.59 %	12.82 %	0.46 %	36.39 %
P33	25802311	99.88 %	3.67 %	18.18 %	11.68 %	0.40 %	36.47 %
P34	27390388	99.84 %	4.59 %	23.77 %	15.12 %	0.34 %	34.83 %
P35	29249396	99.85 %	4.52 %	20.86 %	13.01 %	0.31 %	34.94 %
P36	30457805	99.83 %	4.93 %	20.09 %	12.76 %	0.32 %	33.81 %

Supplementary Table 24. The selected set of interesting core metabolic Kyoto Encyclopedia of Genes and Genomes (KEGG) modules investigated in this study.

**Selected core KEGG modules**
M00001 Glycolysis (Embden-Meyerhof pathway), glucose => pyruvate
M00002 Glycolysis, core module involving three-carbon compounds
M00003 Gluconeogenesis, oxaloacetate => fructose-6P
M00307 Pyruvate oxidation, pyruvate => acetyl-CoA
M00009 Citrate cycle (TCA cycle, Krebs cycle)
M00010 Citrate cycle, first carbon oxidation, oxaloacetate => 2-oxoglutarate
M00011 Citrate cycle, second carbon oxidation, 2-oxoglutarate => oxaloacetate
M00004 Pentose phosphate pathway (Pentose phosphate cycle)
M00006 Pentose phosphate pathway, oxidative phase, glucose 6P => ribulose 5P
M00007 Pentose phosphate pathway, non-oxidative phase, fructose 6P => ribose 5P
M00580 Pentose phosphate pathway, archaea, fructose 6P => ribose 5P
M00005 PRPP biosynthesis, ribose 5P => PRPP
M00008 Entner-Doudoroff pathway, glucose-6P => glyceraldehyde-3P + pyruvate
M00308 Semi-phosphorylative Entner-Doudoroff pathway, gluconate => glycerate-3P
M00633 Semi-phosphorylative Entner-Doudoroff pathway, gluconate/galactonate => glycerate-3P
M00309 Non-phosphorylative Entner-Doudoroff pathway, gluconate/galactonate => glycerate
M00165 Reductive pentose phosphate cycle (Calvin cycle)
M00168 CAM (Crassulacean acid metabolism), dark
M00169 CAM (Crassulacean acid metabolism), light
M00172 C4-dicarboxylic acid cycle, NADP - malic enzyme type
M00171 C4-dicarboxylic acid cycle, NAD - malic enzyme type
M00170 C4-dicarboxylic acid cycle, phosphoenolpyruvate carboxykinase type
M00173 Reductive citrate cycle (Arnon-Buchanan cycle)
M00376 3-Hydroxypropionate bi-cycle
M00375 Hydroxypropionate-hydroxybutylate cycle
M00374 Dicarboxylate-hydroxybutyrate cycle
M00377 Reductive acetyl-CoA pathway (Wood-Ljungdahl pathway)
M00579 Phosphate acetyltransferase-acetate kinase pathway, acetyl-CoA => acetate
M00620 Incomplete reductive citrate cycle, acetyl-CoA => oxoglutarate
M00567 Methanogenesis, CO2 => methane
M00357 Methanogenesis, acetate => methane
M00356 Methanogenesis, methanol => methane
M00563 Methanogenesis, methylamine/dimethylamine/trimethylamine => methane
M00358 Coenzyme M biosynthesis
M00608 2-Oxocarboxylic acid chain extension, 2-oxoglutarate => 2-oxoadipate => 2-oxopimelate => 2-oxosuberate
M00174 Methane oxidation, methanotroph, methane => formaldehyde
M00346 Formaldehyde assimilation, serine pathway
M00345 Formaldehyde assimilation, ribulose monophosphate pathway
M00344 Formaldehyde assimilation, xylulose monophosphate pathway
M00378 F420 biosynthesis, archaea
M00935 Methanofuran biosynthesis
M00422 Acetyl-CoA pathway, CO2 => acetyl-CoA
M00175 Nitrogen fixation, nitrogen => ammonia
M00531 Assimilatory nitrate reduction, nitrate => ammonia
M00530 Dissimilatory nitrate reduction, nitrate => ammonia
M00529 Denitrification, nitrate => nitrogen
M00528 Nitrification, ammonia => nitrite
M00804 Complete nitrification, comammox, ammonia => nitrite => nitrate
M00973 Anammox, nitrite + ammonia => nitrogen
M00176 Assimilatory sulfate reduction, sulfate => H2S
M00596 Dissimilatory sulfate reduction, sulfate => H2S
M00595 Thiosulfate oxidation by SOX complex, thiosulfate => sulfate
